# Metabolic engineering of *Agrobacterium *sp. strain ATCC 31749 for production of an α-Gal epitope

**DOI:** 10.1186/1475-2859-9-1

**Published:** 2010-01-12

**Authors:** Anne M Ruffing, Rachel R Chen

**Affiliations:** 1 School of Chemical and Biomolecular Engineering, Georgia Institute of Technology, 311 Ferst Drive, Atlanta, GA 30332-0100, USA

## Abstract

**Background:**

Oligosaccharides containing a terminal Gal-α1,3-Gal moiety are collectively known as α-Gal epitopes. α-Gal epitopes are integral components of several medical treatments under development, including flu and HIV vaccines as well as cancer treatments. The difficulty associated with synthesizing the α-Gal epitope hinders the development and application of these treatments due to the limited availability and high cost of the α-Gal epitope. This work illustrates the development of a whole-cell biocatalyst for synthesizing the α-Gal epitope, Gal-α1,3-Lac.

**Results:**

*Agrobacterium *sp. ATCC 31749 was engineered to produce Gal-α1,3-Lac by the introduction of a UDP-galactose 4'-epimerase:α1,3-galactosyltransferase fusion enzyme. The engineered *Agrobacterium *synthesized 0.4 g/L of the α-Gal epitope. Additional metabolic engineering efforts addressed the factors limiting α-Gal epitope production, namely the availability of the two substrates, lactose and UDP-glucose. Through expression of a lactose permease, the intracellular lactose concentration increased by 60 to 110%, subsequently leading to an improvement in Gal-α1,3-Lac production. Knockout of the curdlan synthase gene increased UDP-glucose availability by eliminating the consumption of UDP-glucose for synthesis of the curdlan polysaccharide. With these additional engineering efforts, the final engineered strain synthesized approximately 1 g/L of Gal-α1,3-Lac.

**Conclusions:**

The *Agrobacterium *biocatalyst developed in this work synthesizes gram-scale quantities of α-Gal epitope and does not require expensive cofactors or permeabilization, making it a useful biocatalyst for industrial production of the α-Gal epitope. Furthermore, the engineered *Agrobacterium*, with increased lactose uptake and improved UDP-glucose availability, is a promising host for the production of other medically-relevant oligosaccharides.

## Background

α-Gal epitopes are oligosaccharides containing terminal Gal-α1,3-Gal residues. In nature, three main α-Gal epitopes are produced: two trisaccharides (Gal-α1,3-Gal-β1,4-GlcNAc and Gal-α1,3-Lac) and a pentasaccharide (Gal-α1,3-Gal-β1,4-GlcNAc-β1,3-Gal-β1,4-Glc). These epitopes are components of glycolipids and glycoproteins displayed on the cell surface of non-primate mammals and New World monkeys via expression of an α1,3-galactosyltransferase (α1,3-GalT). The α1,3-GalT was inactivated in ancestral Old World primates approximately 20-28 million years ago, resulting in the absence of α-Gal epitopes in humans, apes, and Old World monkeys today [1,2]. These evolutionary descendents of Old World primates produce an antibody to Gal-α1,3-Gal-containing oligosaccharides known as anti-Gal. Anti-Gal is the most abundant natural antibody in humans, and as a result, exposure to α-Gal epitopes generates a strong immune response [3]. Many current research efforts exploit the human immune response to α-Gal epitopes. The efficacy of a vaccine is often determined by uptake of the vaccine by antigen presenting cells. Uptake can be greatly enhanced by the presence of an IgG antibody, such as anti-Gal, bound to its associated antigen. Based on this principle, several vaccines have been modified with α-Gal epitopes in an effort to improve vaccine uptake and efficacy. This strategy was applied to flu and HIV vaccines and was found to be more effective than the non-modified vaccine in animal studies [4,5]. In addition to enhancing vaccine efficacy, the immunogenicity of α-Gal epitopes has been applied to improve cancer treatments. Autologous tumor vaccines with α-Gal epitopes on the tumor cells and injections of α-Gal-containing glycolipids were shown to generate an immune response against malignant tumors in mice [6,7]. The promising results of these α-Gal-based treatments have stimulated the demand for α-Gal epitope production.

The increasing interest in α-Gal epitopes for various medical applications necessitates an efficient and economical means of synthesizing the oligosaccharide. Traditional chemical synthesis requires numerous reaction steps, leading to low overall yields, a high cost, and a process that is not applicable for large-scale production. Enzymatic production of α-Gal epitopes can be achieved in just one step through the use of an α1,3-GalT; however, enzymatic synthesis requires provision of an expensive sugar nucleotide, UDP-galactose. To reduce cost, enzymatic synthesis schemes often employ a UDP-galactose 4'-epimerase to provide the UDP-galactose from a less expensive sugar nucleotide, UDP-glucose [8,9]. As UDP-glucose is still quite expensive, other enzymatic synthesis schemes have been developed to regenerate UDP-galactose through the use of additional enzymes [10,11]. While these synthesis schemes reduce the cost of sugar nucleotide provision, they require production and purification of multiple enzymes, generally 4 to 6, and may also require other high energy compounds such as PEP which can still lead to high synthesis cost.

Alternatively, whole cell biocatalysts can synthesize α-Gal epitopes in just one step without enzyme purification. Different hosts and engineering strategies were explored by Wang and coworkers for whole-cell Gal-α1,3-Lac synthesis. An engineered *E. coli *was constructed by overexpressing five enzymes: three enzymes of the Gal operon (GalK, GalT, GalU) for UDP-galactose synthesis, a pyruvate kinase for energy production, and an α1,3-GalT. In this strategy, glucose and catalytic amounts of other cofactors (ATP, UDPG, and G1P) were supplied to the engineered *E. coli *to initiate Gal-α1,3-Lac synthesis [12]. Alternatively, an engineered *Pichia pastoris *expressed a sucrose synthase which directly converts sucrose and UDP to UDP-glucose with fructose as co-product. With two additional enzymes (UDP-glucose 4'-epimerase and α1,3-GalT), the modified *P. pastoris *required only sucrose, lactose, a catalytic amount of UDP, and a few essential nutrients to produce Gal-α1,3-Lac. The three recombinant enzymes in *P. pastoris *constituted an artificial pathway, whose operation was independent of cellular metabolism [13]. Both the engineered *E. coli *and *P. pastoris *were capable of synthesizing gram-scale amounts of Gal-α1,3-Lac.

One key challenge with whole-cell catalysts is uptake of the acceptor sugar, lactose, along with the primary sugar (i.e. sucrose or glucose). In both the engineered *E. coli *and *P. pastoris *biocatalysts, permeabilization was found to be necessary [12,13]. While effective at improving lactose uptake, permeabilization also reduces cell viability and dissipates the proton gradient used for energy generation. This may not be a problem when cellular metabolism is not required to generate UDP-glucose, such is the case with sucrose synthase, but otherwise, it is detrimental to both cellular metabolism and α-Gal epitope synthesis. To avoid permeabilization, an *E. coli *biocatalyst was engineered to synthesize the acceptor sugar *in vivo*. Expressing a chitin oligosaccharide synthase (NodC) and several glycosyltransferases, the recombinant *E. coli *produced a heptasaccharide α-Gal epitope without permeabilization of the cell membrane [14]. While this method avoids the complications imposed by multiple sugar uptake, the diversion of cell resources for *in vivo *substrate synthesis may hinder α-Gal epitope production.

**Figure 1:**
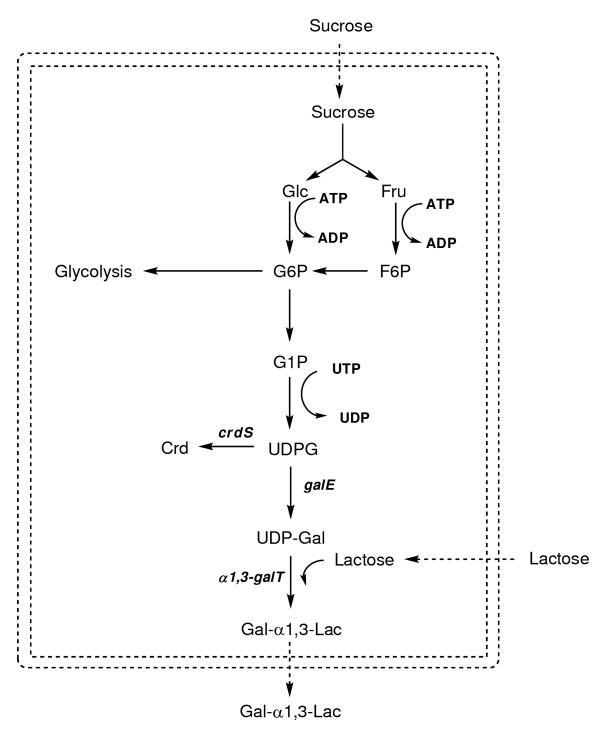
**Metabolic pathway for Gal-α1,3-Lac synthesis**. Metabolic pathways of ATCC 31749 for Gal-α1,3-Lac synthesis. Cofactors are shown in bold and enzymes are in bold italics. The double dashed lines indicate the cell membrane, and the dashed arrows represent transport reactions.

We have previously demonstrated that *Agrobacterium *sp. strain ATCC 31749 is a good host for oligosaccharide production through the synthesis of β1,4-Gal disaccharides [15]. ATCC 31749 naturally produces high amounts of a β1,3-glucan polysaccharide known as curdlan. High curdlan production in ATCC 31749 implies an efficient mechanism for regeneration of the requisite sugar nucleotide, UDP-glucose. In our previous work, ATCC 31749 was engineered to convert the UDP-glucose regeneration system into a UDP-galactose regeneration system. This current study utilizes the efficient UDP-galactose production for synthesis of the medically-relevant α-Gal epitope, Gal-α1,3-Lac. Instead of using permeabilization or *in vivo *substrate synthesis, an alternative strategy is employed to address insufficient uptake of the substrate sugar, lactose. A lactose permease (LacY) from *E. coli *is introduced in the *Agrobacterium *host to facilitate lactose transport across the cell membrane. In addition, sugar nucleotide availability is improved by eliminating curdlan production, a competing pathway for utilization of UDP-glucose (Figure 1).

## Results

### α-Gal epitope synthesis using ATCC 31749/pBQET

**Figure 2:**
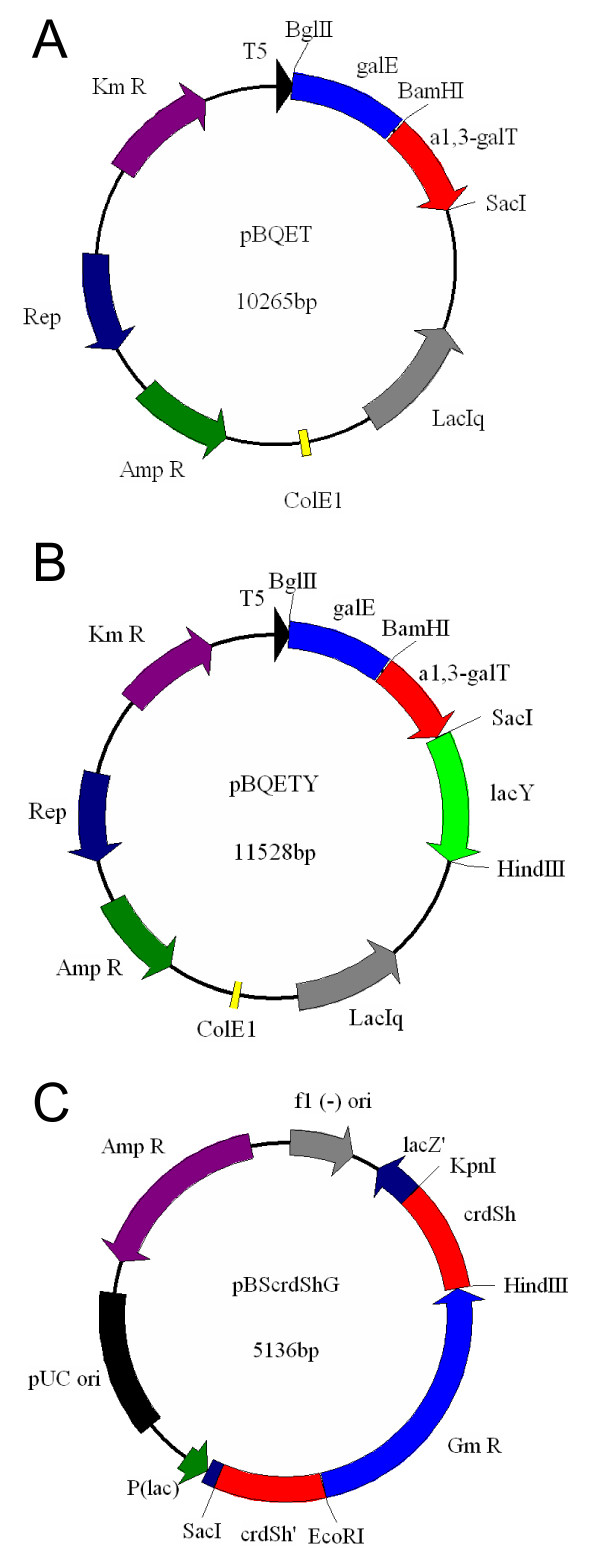
**Plasmids constructed in this study**. A: pBQET, containing the *galE:α1,3-galT *fusion enzyme for Gal-α1,3-Lac synthesis; B: pBQETY, containing the *galE:α1,3-galT *fusion enzyme and *lacY *for Gal-α1,3-Lac synthesis and lactose transport; C: pBScrdShG, containing homologous regions of *crdS *interrupted by a gentamicin resistance cassette for *crdS *knockout.

ATCC 31749 naturally produces high amounts of curdlan polysaccharide, suggesting an abundant supply of the sugar nucleotide precursor, UDP-glucose. In order to utilize UDP-glucose for synthesis of Gal-α1,3-Lac, two additional enzymes are required: a UDP-galactose 4'-epimerase and an α1,3-galactosyltransferase (Figure 1). In previous work, we demonstrated that the expression of a *galE:lgtB *fusion gene in ATCC 31749 can efficiently produce β1,4-Gal oligosaccharides [15]. Adopting a similar strategy for α-Gal epitope synthesis, a *galE:α1,3-galT *fusion gene was constructed using a UDP-galactose 4'-epimerase (*galE*) from *E. coli *and a truncated bovine α1,3-galactosyltransferase (*α1,3-galT*) (Figure 2A). Inserting the fusion gene into the *Agrobacterium *expression vector, pBQ, yielded pBQET, which was transformed into ATCC 31749 for synthesis of the α-Gal epitope. Successful expression of the GalE:α1,3-GalT fusion enzyme was confirmed with an enzyme activity assay using ATCC 31749/pBQET. Concentrations of IPTG ranging from 0 to 1 mM were investigated to maximize activity of the fusion enzyme in the ATCC 31749 host. From 0 to 0.5 mM IPTG, the fusion enzyme activity increased with increasing IPTG concentration and reached a plateau from 0.5 to 1 mM IPTG (Table 1). A concentration of 1 mM IPTG was selected for all subsequent α-Gal epitope synthesis reactions.

**Table 1:** GalE:α1,3-GalT fusion enzyme activity^†^

Strain	IPTG (mM)	Enzyme Activity [μmol/(min*L)]
ATCC 31749/pBQET	0	1.2 ± 0.76
	0.05	10.7 ± 1.1
	0.1	28.0 ± 0.52
	0.5	53.4 ± 16.3
	1	54.1 ± 5.3
		
ATCC 31749/pBQETY	0	6.4 ± 0.96
	0.05	13.2 ± 0.058
	0.1	15.6 ± 1.6
	0.5	14.3 ± 0.72
	1	11.6 ± 1.2
		
ATCC 31749Δ*crdS*/pBQET	1	38.2 ± 1.7
		
ATCC 31749Δ*crdS*/pBQETY	1	12.6 ± 0.79

^†^Enzyme activity of GalE:α1,3-GalT fusion enzyme with varying IPTG concentration. Data are reported as averages of at least two independent experiments with the associated standard deviation serving as an estimation of error.

**Figure 3:**
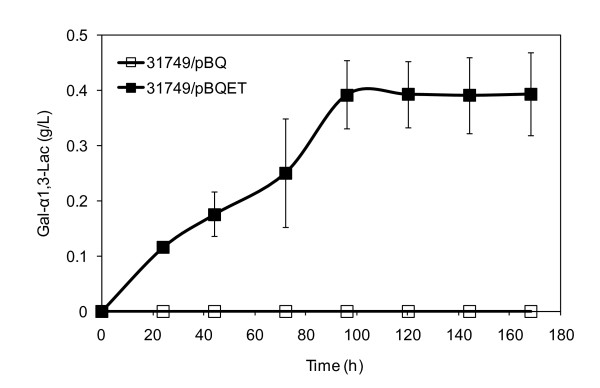
**Gal-α1,3-Lac synthesis by ATCC 31749/pBQET**. Synthesis of Gal-α1,3-Lac by ATCC 31749/pBQ (control) and ATCC 31749/pBQET. Data points are averages of three independent experiments with the standard deviation indicated by error bars.

The engineered *Agrobacterium*, ATCC 31749/pBQET, was utilized for small-scale α-Gal epitope synthesis. The synthesis process includes two phases. In the first phase, the engineered *Agrobacterium *is grown and induced. After recombinant protein production, the cells are transferred to a nitrogen-limited minimal media for synthesis of Gal-α1,3-Lac. Nitrogen-limited conditions are employed for α-Gal epitope synthesis as curdlan production, and hence UDP-glucose production, is activated by this environmental signal. In the α-Gal epitope synthesis reaction, the engineered strain synthesized 0.39 g/L of the desired product, Gal-α1,3-Lac (Figure 3). While this result confirms the successful engineering of ATCC 31749 for α-Gal epitope synthesis, the concentration of Gal-α1,3-Lac produced is lower than expected. In our previous work, the engineered *Agrobacterium *synthesized up to 7.5 g/L of β1,4-Gal oligosaccharides, nearly 20-fold greater than the amount of Gal-α1,3-Lac produced by ATCC 31749/pBQET [15]. Since ATCC 31749 has the potential to produce much higher levels of the α-Gal epitope, additional metabolic engineering strategies were investigated, focusing mainly on increased acceptor uptake and UDP-glucose availability.

### Increased uptake of the acceptor, lactose

A major challenge of whole-cell synthesis is the efficient transport of reactants across the cell membrane. This task is particularly difficult for oligosaccharide synthesis reactions such as α-Gal epitope synthesis. The synthesis reaction requires uptake of two sugars: the acceptor sugar (lactose) and a carbon source for production of the sugar nucleotide and cellular energy (sucrose) (Figure 1). Simultaneous uptake of multiple sugars is often prohibited by catabolite repression systems which allow for uptake of only a preferred carbon source [16]. Nominal uptake of the acceptor, lactose, was suspected to limit synthesis of the α-Gal epitope in ATCC 31749/pBQET. Measurement of lactose uptake by ATCC 31749 supported this hypothesis, as the rate of lactose consumption was more than 45-fold lower than the rate of sucrose consumption. To confirm that lactose is limiting for Gal-α1,3-Lac synthesis, the extracellular concentration of lactose was increased from 25 g/L to 50 g/L in an effort to increase the intracellular lactose concentration by means of diffusion. The higher lactose concentration led to a 28% increase in Gal-α1,3-Lac synthesis. An estimate of the intracellular lactose concentration indicates that roughly 1 mM of lactose is present intracellularly, which is more than 8-fold lower than the reported K_m _for the GalE:LgtB fusion enzyme (K_m _= 8.5 mM) [17]. These results indicate that lactose availability may limit α-Gal epitope synthesis.

While doubling the concentration of lactose was successful at increasing α-Gal epitope production, diffusion-mediated transport across the cell membrane is limited, requiring a large increase in lactose concentration to bring about a small improvement in synthesis. In this work, an alternative strategy is explored: introducing a heterologous lactose transporter to increase the availability of intracellular lactose. A lactose permease gene (*lacY*) from an *E. coli *K12 strain was expressed along with the fusion enzyme in the engineered *Agrobacterium *(Figure 2B). LacY is a lactose/proton symporter that transports lactose across the cytoplasmic membrane [18]. As expression of a transmembrane protein is generally not as straightforward as other soluble proteins, additional steps were taken to provide evidence of successful expression of *lacY*. To analyze the expression and activity of LacY in the engineered *Agrobacterium*, uptake of lactose by ATCC 31749/pBQETY was compared to the lactose uptake of ATCC 31749/pBQET through analysis of residual lactose concentration in the extracellular medium. The rate of lactose uptake in ATCC 31749/pBQETY was approximately 50% greater than that of ATCC 31749/pBQET, indicating successful expression and activity of LacY. This measurement relies on extracellular lactose measurement, however, which assumes that decreasing lactose concentration is due solely to uptake by the cell, with no degradation of lactose in the extracellular medium. To confirm that LacY expression does indeed enhance lactose uptake, the amount of intracellular lactose was directly measured throughout the time course of the α-Gal synthesis reaction. These measurements show a 60 to 110% increase in intracellular lactose concentration for ATCC 31749/pBQETY compared to ATCC 31749/pBQET. Taken together, these results suggest successful expression of LacY and functional insertion of the protein into the cytoplasmic membrane.

**Figure 4:**
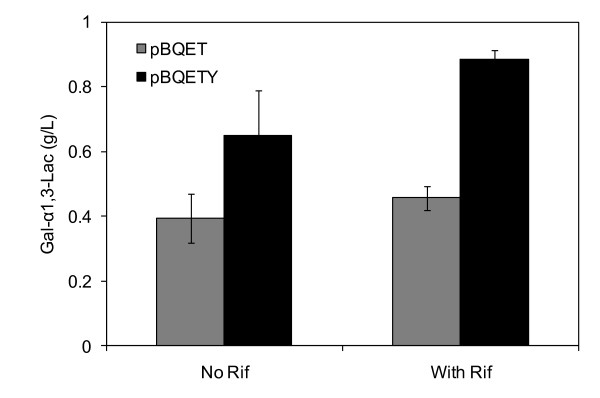
**Comparison of Gal-α1,3-Lac synthesis by ATCC 31749/pBQET and ATCC 31749/pBQETY**. Synthesis of Gal-α1,3-Lac after 150 hours by ATCC 31749/pBQET and ATCC 31749/pBQETY without and with rifampicin. Data are averages of three independent experiments with the standard deviation indicated by error bars.

ATCC 31749/pBQETY was employed to determine the effect of increased lactose availability on α-Gal epitope synthesis. The LacY-expressing strain synthesized 0.65 g/L of Gal-α1,3-Lac, a 67% improvement over the initial engineered strain, ATCC 31749/pBQET (Figure 4). Surprisingly, the activity of the GalE:α1,3-GalT fusion enzyme was 4.7-fold lower in ATCC 31749/pBQETY compared to ATCC 31749/pBQET. The lower activity in the LacY-expressing strain is presumably due to lower expression of the fusion enzyme. Unlike ATCC 31749/pBQET, the expression exhibited weak dependence on IPTG concentration (Table 1). To determine if the lower fusion enzyme activity of ATCC 31749/pBQETY restricts Gal-α1,3-Lac production, 0.05 mM of IPTG was used for α-Gal epitope synthesis, as both ATCC 31749/pBQET and ATCC 31749/pBQETY have similar fusion enzyme activities at this IPTG concentration. The amount of Gal-α1,3-Lac synthesized by the LacY-expressing strain was similar to that produced with 1 mM IPTG; this was expected as the enzyme activity levels are very similar at both IPTG concentrations. Unexpectedly, the amount of Gal-α1,3-Lac produced by ATCC 31749/pBQET was similar for both 0.05 mM and 1 mM IPTG despite over a 5-fold reduction in fusion enzyme activity (Table 1). This suggests that the activity of the fusion enzyme is not a limiting factor in Gal-α1,3-Lac synthesis in the wild type background.

### Curdlan synthase knockout for improved UDP-glucose availability

In addition to limitations imposed by low acceptor (lactose) availability, the amount of the sugar nucleotide, UDP-glucose, may also restrict α-Gal epitope synthesis by the GalE:α1,3-GalT fusion enzyme. Under the nitrogen-limited conditions of the synthesis reaction, production of the α-Gal epitope competes with curdlan synthesis for the available UDP-glucose (Figure 1). To determine if UDP-glucose availability limits Gal-α1,3-Lac production, rifampicin was added to the synthesis reaction to prevent curdlan production. Since curdlan is only produced under nitrogen-limitation [19], it is suspected that the curdlan synthesis operon is only transcribed after exhaustion of the nitrogen source. Therefore, rifampicin, a transcription inhibitor, should prevent transcription of the genes responsible for curdlan synthesis during the production of Gal-α1,3-Lac. The addition of rifampicin led to a 37% increase in Gal-α1,3-Lac synthesis in ATCC 31749/pBQETY (Figure 4), indicating that UDP-glucose availability may limit α-Gal epitope production.

Curdlan, a β1,3-glucan polysaccharide, is not known to perform any essential or beneficial function for ATCC 31749. Therefore, by eliminating curdlan production, the amount of UDP-glucose available for α-Gal epitope synthesis will be increased without any detrimental impact on the cell. The gene responsible for the transfer of glucose from UDP-glucose to the growing curdlan polymer chain was previously determined to be curdlan synthase, c*rdS *[20]. CrdS has been characterized and the nucleotide sequence reported, providing the necessary information required for gene knockout.

**Figure 5:**
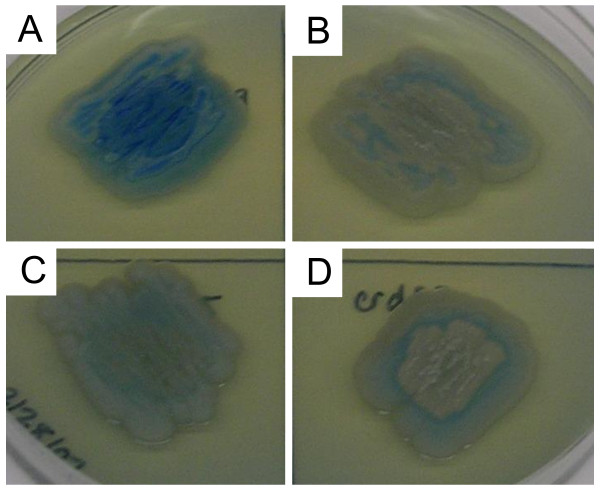
**Aniline blue staining of curdlan**. Aniline blue staining of curdlan production in wild-type and *crdS *mutant strains. A: ATCC 31749; B: ATCC 31749ΔcrdS colony 8; C: LTU265; D: ATCC 31749ΔcrdS colony 14.

This is the first reported attempt at gene knockout in *Agrobacterium *sp. strain ATCC 31749; however, successful gene knockout has been reported for a related organism, *Agrobacterium tumefaciens*. Gene knockout in *A. tumefaciens *employed the standard method of insertional mutagenesis via homologous recombination of a disruption cassette [21]. To determine an effective antibiotic resistance cassette for gene knockout, ATCC 31749 was tested for resistance against several antibiotics. Two antibiotics, gentamicin and tetracycline, were found to be effective at preventing ATCC 31749 growth at concentrations greater than 50 μg/mL. Since the genome of *A. tumefaciens *contains several tetracycline resistance genes, gentamicin was selected for the antibiotic resistance cassette in the *crdS *knockout plasmid. Sequences homologous to the 5' and 3' ends of *crdS *(500 bp) were added to each side of the gentamicin resistance cassette to allow for homologous recombination. The resulting fragment was inserted into the pBluescript II (KS^-^) phagemid to give the *crdS *knockout plasmid, pBScrdShG (Figure 2C). After transformation of the *crdS *knockout plasmid into ATCC 31749, gentamicin-resistant colonies were screened using PCR for the presence of a 2236 kb fragment containing the interrupted *crdS *with gentamicin resistance cassette and the absence of the 1965 kb fragment corresponding to the intact *crdS*. A functional screening was also performed to confirm successful disruption of *crdS*, using aniline blue staining to detect curdlan production. The aniline blue dye binds to β1,3-glucan linkages in curdlan and produces a bright blue color [22]. In Figure 5, two candidate *crdS *mutants are compared to the wild type ATCC 31749 strain and a curdlan-deficient mutant produced via NTG mutagenesis, LTU265. The two *crdS *mutants show only a faint blue color, similar to LTU265, indicating curdlan synthesis was drastically reduced. As additional evidence for successful c*rdS *knockout, ATCC 31749Δ*crdS *was tested for the formation of curdlan gel. Heating an aqueous curdlan solution above 80°C and cooling will lead to the formation of a curdlan gel [23]. The *crdS *mutant strain was cultivated in nitrogen-limited media to initiate curdlan production, yet the mutant did not form any visible curdlan gel after the requisite heating and cooling (data not shown). The cell growth profile for ATCC 31749Δ*crdS *in LB media showed no deviation from the wild-type strain, indicating that the *crdS *knockout did not affect cell growth. Since curdlan is not produced during cell growth, but rather, under nitrogen-limited conditions, the cell viability of ATCC 31749Δ*crdS *was studied in minimal, nitrogen-free media. The cell viability of the *crdS *mutant under curdlan-producing conditions showed no significant difference from the wild-type strain (data not shown). Therefore, curdlan production does not appear to contribute to cell survival under nitrogen-limited conditions and should not have a detrimental effect on α-Gal epitope synthesis.

**Figure 6:**
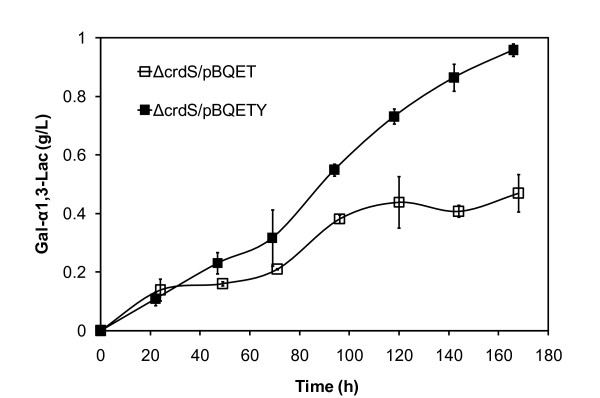
**Gal-α1,3-Lac synthesis by the engineered *crdS *mutants**. Synthesis of Gal-α1,3-Lac by ATCC 31749Δ*crdS*/pBQET and ATCC 31749Δ*crdS*/pBQETY. Data points are averages of three independent experiments with the standard deviation indicated by error bars.

To determine the effect of the curdlan synthase knockout on α-Gal epitope synthesis, ATCC 31749Δ*crdS *was transformed with pBQET and pBQETY. ATCC 31749Δ*crdS*/pBQETY produced 0.96 g/L of Gal-α1,3-Lac (Figure 6), a concentration similar to that achieved with the addition of rifampicin (Figure 4). On the other hand, ATCC 31749Δ*crdS*/pBQET synthesized only 0.40 g/L of the α-Gal epitope, indicating that synthesis remains limited by lactose availability. Overall, both lactose and UDP-glucose availability contributed to the low levels of Gal-α1,3-Lac produced by the initial engineered strain, ATCC 31749/pBQET. The construction of ATCC 31749Δ*crdS*/pBQETY, with increased levels of intracellular lactose and UDP-glucose, demonstrates the potential of ATCC 31749 as host for production of the α-Gal epitope. Requiring only a carbon source, the acceptor sugar, and a few essential nutrients (a phosphate source, buffer, and metal cofactors), the engineered *Agrobacterium *is capable of producing gram-scale quantities of the α-Gal epitope for medical research and applications.

## Conclusions and Discussion

The whole-cell biocatalysts developed in this study provide a basis for an efficient and cost-effective means for large-scale production of the Gal-α1,3-Lac epitope. Enzymatic and whole-cell methods developed for α-Gal epitope synthesis face many obstacles including (1) low synthesis levels, (2) inefficient substrate transport across the cell membrane, and (3) expensive cofactors. This work addresses all three of these issues. Host selection is a critical factor in determining product synthesis levels. To provide an adequate host for synthesis of the oligosaccharide, Gal-α1,3-Lac, a polysaccharide-producing microorganism was selected, *Agrobacterium *sp. ATCC 31749. This microorganism can produce up to 93 g/L of curdlan polysaccharide [24], demonstrating a natural proclivity for synthesizing the sugar nucleotide precursor, UDP-glucose. With ATCC 31749 as host, gram-scale quantities of Gal-α1,3-Lac were produced, demonstrating an advantage over traditional hosts such as *E. coli*. Insufficient uptake of substrate and acceptor sugars is yet another obstacle in whole-cell oligosaccharide synthesis. To enhance sugar uptake, other strategies have used permeabilization techniques to weaken the cell membrane or have expressed additional enzymes to synthesize the acceptor sugar *in vivo *[12-14]. In this work, a lactose permease was introduced into the *Agrobacterium *host to selectively increase uptake of the acceptor, lactose. This strategy increased lactose uptake without the undesirable consequences of cell permeabilization techniques, leading to a 67% improvement in Gal-α1,3-Lac synthesis. The LacY-expressing *Agrobacterium *may also be used to synthesize other lactose-containing oligosaccharides such as Globo-H, the major component of a vaccine for metastatic breast cancer [25]. Lastly, expensive cofactors are often added to enhance production of the sugar nucleotide, including UDP, ATP, PEP, or even UDP-glucose itself. By deleting the gene responsible for curdlan synthesis, the efficient UDP-glucose synthesis pathway of the *Agrobacterium *host was exploited for α-Gal epitope synthesis, negating a need for expensive cofactors. Without the requirements of cell permeabilization or cofactors, the engineered *Agrobacterium *developed in this work is a suitable biocatalyst for large-scale production of the α-Gal epitope, and with only slight modification, it may be utilized for the production of other medically-relevant oligosaccharides.

While the results clearly show that the metabolic engineering strategies of *lacY *expression and *crdS *knockout are effective in addressing the two respective limitations, these efforts resulted in only moderate improvements in α-Gal epitope synthesis. The highest product concentration was about 1 g/L with shaker flask cultivation, far below the theoretical potential of 270 g/L (estimated from reported levels of curdlan synthesis in *Agrobacterium *sp.). One reason for the lower than expected improvement is that LacY expression negatively impacted expression of the fusion enzyme. As shown in Table 1, the addition of *lacY *to pBQET leads to nearly a 5-fold reduction in GalE:α1,3-GalT fusion enzyme activity. In the future, chromosomal integration of *lacY *may be considered to improve lactose uptake while maintaining the high fusion enzyme activity of the pBQET strain. The use of shaker flask cultivation may also contribute to the low level of Gal-α1,3-Lac synthesis. Other reported methods of whole-cell α-Gal epitope synthesis utilize fermenters for either high cell density growth or both growth and the epitope synthesis reaction [12-14]. Large-scale fermentation may be particularly beneficial with the engineered *Agrobacterium *as high curdlan synthesis requires high levels of dissolved oxygen and pH control at pH 5.5 [26,27]. Under these conditions, the engineered *crdS *mutant should produce high levels of UDP-glucose for Gal-α1,3-Lac synthesis. Unfortunately, initial fermentation attempts with the engineered *Agrobacterium *were unsuccessful due to the low recombinant protein production. Chromosomal integration of the *galE:α1,3galT *fusion enzyme and expression using a natural host promoter may overcome the limited recombinant protein production to allow large-scale fermentation. The reasons for only modest improvement from *crdS *knockout are not clear to us at this point due to limited knowledge about the regulation of curdlan synthesis. Recently, we sequenced the genome of ATCC 31749 and a transcriptome analysis is being conducted. These efforts will lead to a better understanding of the regulation mechanism governing curdlan synthesis and its precursor, UDP-glucose. In turn, this knowledge will allow metabolic engineers to formulate better strategies for improving synthesis of α-Gal epitopes and other oligosaccharides using ATCC 31749 as host.

## Methods

### Materials

The chemicals used in this study were obtained from Sigma-Aldrich (lactose, K_2_HPO_4_·3H_2_O, and MnCl_2_·4H_2_O); Acros organics (aniline blue); V-labs, Inc. (Gal-α1,3-Gal-β1,4-Glc); and Fisher (all other chemicals).

### Bacterial strains and plasmids

The bacterial strains and plasmids used in this study are described in Table 2.

**Table 2:** Bacterial strains and plasmids

Strains or plasmids	Description	Source
ATCC 31749	Curdlan-producing *Agrobacterium *sp.	ATCC
LTU265	ATCC 31749 locus II mutant with decreased curdlan production	B.A. Stone V.A. Stanisich
JM109	*E. coli *K12 strain used for *lacY *cloning	Promega
ATCC 31749Δ*crdS*	ATCC 31749 with interrupted curdlan synthase gene (c*rdS*)	This study
pGEM-T easy	Vector used for gene cloning	Promega
pBQ	Broad-host-range expression vector constructed for gene expression in ATCC 31749	[15]
pET15b-αGalT	Plasmid containing truncated bovine α1,3-galactosyltransferase gene	[11]
pT-α1,3-galT	pGEM-T easy vector containing truncated bovine α1,3-galactosyltransferase with *BamHI *and *SacI *restriction sites	This study
pT-GalE	Plasmid containing UDP-galactose 4'-epimerase (*galE*) from *E. coli *with a short linker sequence for fusion enzyme construction	[15]
pTET	pGEM-T easy vector containing the *gale:α1,3galT *fusion gene	This study
pBQET	Plasmid for GalE:α1,3GalT fusion enzyme expression in ATCC 31749 and ATCC 31749Δ*crdS*	This study
pT-lacY	pGEM-T easy vector containing lactose permease gene (*lacY*) from JM109 with *SacI *and *XhoI *restriction sites	This study
pBQETY	Plasmid for expression of GalE:α1,3GalT fusion enzyme and *E. coli *lactose permease (LacY) in ATCC 31749 and ATCC 31749Δ*crdS*	This study
pT-crdS	pGEM-T easy vector containing curdlan synthase gene (c*rdS*) with *SacI *and *KpnI *restriction sites	This study
pT-crdSh	pGEM-T easy vector containing two 500 bp regions of homology to *crdS *with *SacI/KpnI *and *EcoRI*/*HindIII *restriction sites	This study
pBluescript II (KS^-^)	Phagemid for gene knockout in ATCC 31749	Stratagene
pBScrdSh	pBluescript II (KS^-^) containing two 500 bp regions of homology to *crdS *with *EcoRI *and *HindIII *restriction sites	This study
pYanni2	Plasmid containing a gentamicin resistance cassette	[28]
pT-GmR	pGEM-T easy vector containing gentamicin resistance cassette with *EcoRI *and *HindIII *restriction sites	This study
pBScrdShG	*crdS *knockout plasmid; pBluescript II (KS^-^) containing a gentamicin resistance cassette flanked by two 500 bp regions of homology to *crdS*	This study

### Construction of pBQET and pBQETY for α-Gal epitope synthesis

A fusion enzyme containing the *E. coli *UDP-galactose 4'-epimerase (*galE*) and truncated bovine α1,3-galactosyltransferase (*α1,3-galT*) was constructed and inserted into the *Agrobacterium *expression vector, pBQ, to form pBQET for α-Gal epitope synthesis. The truncated bovine *α1,3-galT *was amplified from pET15b-αGalT [11]. The 5' primer for *α1,3-galT *(5'-TC***GGATCC***ATGGAAAGCAAGCTTAAGCTATC-3') contains a *BamHI *site (in bold italics) and a start codon (underlined). The 3' primer for *α1,3-galT *(5'-TC***GAGCTC***TCAGACATTATTTCTAACCACATT-3') contains a *SacI *site (in bold italics) and a stop codon (underlined). The amplified *α1,3-galT *was inserted into the pGEM-T easy vector to form pT-α1,3-galT. The α1,3-galT fragment, obtained by double digestion with *BamHI *and *SacI*, was inserted into the respective restriction sites of the pT-GalE plasmid containing *galE *with a linker sequence [15]. Successful ligation of the α1,3-galT fragment to the digested pT-GalE yielded pTET, containing the *galE:α1,3-galT *fusion gene for α-Gal epitope synthesis. The fusion gene fragment, obtained from *BglII *and *SacI *digestions, was fused to the *BamHI *and *SacI *sites of pBQ. The resulting plasmid, pBQET, is shown in Figure 2A.

The lactose permease gene (*lacY*) was cloned from the genomic DNA of *E. coli *K12 strain JM109. The 5' primer for *lacY *(5'-AC***GAGCTC***AAAGAGGAGAAAT TAACT**ATG**TACTATTTAAAAAACACAAAC-3') contains a *SacI *site (in bold italics), a ribosome binding site (underlined), and a start codon (in bold and underlined). The 3' primer (5'-GT***CTCGAG***TTAAGCGACTTCATTCACCTG-3') contains an *XhoI *site (in bold italics) and stop codon (underlined). The amplified *lacY *fragment was inserted into the pGEM-T easy vector, yielding pT-LacY. The *lacY *fragment, obtained by *SacI *and *XhoI *digestions, was ligated to the *SacI *and *SalI *sites of pBQET to form pBQETY (Figure 2B).

### Gene knockout of curdlan synthase (*crdS*) in ATCC 31749

The knockout plasmid for gene knockout of the curdlan synthase gene, *crdS*, was constructed using a gentamycin resistance cassette flanked on each side by 500 bp of homology to the curdlan synthase gene. The 5' primer for *crdS *amplification (5'-CG***GA GCTC***ATGTATTTCAGTGCTGAAGG-3') contains a *SacI *site (in bold italics) and the start codon for *crdS *(underlined). The 3' primer for *crdS *amplification (5'-CC***GGTACC***TCACCCGAATGCCCGTGC-3') contains a *KpnI *site (in bold italics) and *crdS *stop codon (underlined). The amplified *crdS *gene was inserted into the pGEM-T easy vector to produce pT-crdS. Using pT-crdS as template, the pGEM-T easy vector along with 500 bp of homology at the 5' end of *crdS *and 500 bp of homology at the 3' end of *crdS *were amplified using phosphorylated primers. The 5' primer (5'-pGGCC***GAATTC***TCGCAATAGGTTCTTACCTC-3') contains an *EcoRI *site (in bold italics) while the 3' primer (5'-pGGC***AAGCTT***TTCGTGACCCTGTCTTCGGC-3') contains a *HindIII *site. The amplified pGEM-T easy vector with homologous regions of *crdS *was self-ligated to form pT-crdSh. pT-crdSh was digested using SacI and KpnI, and the resulting fragment containing *crdS *homology (*crdSh*) was inserted into the corresponding restriction sites of pBluescriptII (KS^-^), generating the plasmid pBScrdSh. The gentamicin resistance gene (*Gm*^*R*^), along with its promoter and transcription terminator, was amplified from pYanni2 [28]. The 5' primer for *Gm*^*R *^amplification (5'-CC***GAATTC***GTCTAGTGAGTAGTG GGTAC-3') contains an *EcoRI *site (in bold italics), and the 3' primer (5'-CG***AAGCTT ***GCTTGCAAACAAAAAAACCACC-3') contains a *HindIII *site. The amplified fragment containing *Gm*^*R *^was inserted into the pGEM-T easy vector, forming pT-GmR. pT-GmR was digested using EcoRI and HindIII, and the fragment containing *Gm*^*R *^was inserted into the corresponding restriction sites in pBScrdSh to form pBScrdShG (Figure 2C). The knockout plasmid, pBScrdShG, was transformed into ATCC 31749 using electroporation, followed by a 5 hour cultivation without antibiotics, and growth on LB/agar plates containing 50 μg/mL of gentamicin. The KO candidate colonies were grown four times in LB media at 30°C and 250 rpm to cure pBScrdShG.

### Cell growth and GalE:α1,3GalT enzyme activity assay

ATCC 31749/pBQET, ATCC 31749/pBQETY, ATCC 31749Δ*crdS*/pBQET, and ATCC 31749Δ*crdS*/pBQETY inoculums were prepared at 30°C and 250 rpm in a culture tube with 4 mL of LB media containing 100 μg/mL of kanamycin and 100 μg/mL of ampicillin. The inoculums were diluted 500× and grown in 150 mL of freshly prepared LB media supplemented with antibiotics until an OD_600 _of 0.3 - 0.4 was reached, upon which IPTG was added to a final concentration of 1 mM. After IPTG addition, the cell culture was incubated at 30°C and 250 rpm for another 6 hours.

For the enzyme activity assay, the induced cells were collected by centrifugation at 5,000 × g and 4°C for 2 min. The cell pellet was resuspended in a buffer containing 25 mM Tris-HCl (pH 7.5), 10 mM MnCl_2_, 100 mM NaCl, and 0.25% Triton X to a final cell concentration of 20% wet wt/v. Using a Branson Mode 250 Sonifer, the mixture was sonicated 6 times for 15 second intervals with 1 min rest on ice. The lysates were centrifuged for 2 min at 10,000 × g at 4°C, and 50 μL of supernatant was added to the substrate mixture for the enzyme activity assay. The substrate mixture consists of 20 mM lactose (10 mM final concentration) and 8 mM of UDP-glucose (4 mM final concentration) in the enzyme activity buffer described previously. The assay was conducted at 30°C for 40 min followed by 10 min at 97°C to stop the reaction. The deactivated enzyme mixture was diluted 50×, and the Gal-α1,3-Lac product was analyzed using the protocol detailed below (Carbohydrate analysis).

### Cell viability

ATCC 31749 and ATCC 31749Δ*crdS *inoculums were prepared at 30°C and 250 rpm in a culture tube with 4 mL of LB media containing 10 μg/mL of gentamicin for the *crdS *mutant. The inoculums were diluted 1000× and grown in 150 mL of freshly prepared LB media, supplemented with antibiotics for ATCC 31749Δ*crdS*. After 12 hours of growth in LB, the cells were collected by centrifugation at 3000 × g for 10 min at 4°C. The cell pellets were washed with 10% glycerol and then resuspended in nitrogen-free media. The nitrogen-free media consisted of 70 g/L sucrose, 1 g/L K_2_HPO_4_·3H_2_O, 5 g/L MgSO_4_·7H_2_O, 5 g/L sodium citrate, 1 g/L MnCl_2_·4H_2_O, and 50 mM Tris-HCl (pH 7.5). The cells were cultivated in nitrogen-free media at 30°C and 250 rpm in a biological shaker for 72 hours. Samples were taken at intervals of 12 hours and diluted using sterile LB media. The diluted cultures were spread on LB/agar plates, and colony forming units (cfu's) were counted as a measure of cell viability.

### α-Gal epitope synthesis

The induced cells were prepared as described above (Cell growth and GalE:α1,3-GalT enzyme activity assay). The cells were then collected by centrifugation for 10 min at 3,000 × g and 4°C. The cell pellets were washed once with sterile 10% glycerol, and then resuspended in the reaction media. The reaction media contained 50 g/L sucrose, 25 g/L lactose, 1 g/L K_2_HPO_4_·3H_2_O, 5 g/L MgSO_4_·7H_2_O, 5 g/L sodium citrate, 1 g/L MnCl_2_·4H_2_O, and 50 mM Tris-HCl (pH 7.5). The final cell concentration was 10% wet wt/v, and the reaction vessel was a 50 mL Erlenmeyer flask. The reaction vessel was placed in a biological shaker at 30°C and 250 rpm. Samples were centrifuged for 3 min at 13,200 rpm. The supernatant was heated in boiling water for 10 min and then centrifuged again at 13,200 rpm for 3 min. The supernatant was appropriately diluted and analyzed as described in Carbohydrate analysis.

### Analytical techniques

#### SDS-PAGE

SDS-PAGE was used to confirm the successful expression of the GalE:α1,3-GalT fusion enzyme. JM109/pBQET was inoculated in 4 mL of LB media containing 100 μg/mL of kanamycin and 100 μg/mL of ampicillin. After overnight growth at 37°C with 250 rpm, 3 mL of the inoculum was transferred to 150 mL of LB media with antibiotics and grown at 37°C with 250 rpm. When an OD_600 _of 0.2 - 0.3 was reached, the cultures were induced with IPTG (final concentration 1 mM). Immediately after the addition of IPTG, the temperature was shifted to 25°C. After an induction period of 4 hours, the culture was centrifuged at 3,000 × g and 4°C for 10 min. The cell pellet was washed with 0.4 M NaCl and collected by centrifugation. A buffer containing 100 mM Tris-HCl and 100 mM NaCl (pH 8.0) with protease inhibitor cocktail (Sigma-Aldrich) was used to resuspend the cell pellet to a final concentration of 0.2 g/mL (wet weight). The cell suspension was sonicated 8 times for 10 second intervals with 1 min rest on ice. Cellular debris was removed by centrifugation at 3,000 × g and 4°C for 20 min. The GalE:α1,3-GalT fusion enzyme was purified from the supernatant using a HIS select HF nickel affinity gel (Sigma-Aldrich). A 12% Tris-HCl gel (Bio-Rad) was used for SDS-PAGE to analyze the His-tag purified and soluble protein fractions.

#### Carbohydrate analysis

Diluted samples were analyzed using a Dionex BioLC system with a CarboPac PA20 analytical column. The Dionex ED50 electrochemical detector measured carbohydrate concentrations through pulsed amperometry (waveform: t = 0.41 sec, p = -2.00 V; t = 0.42 sec, p = -2.00 V; t = 0.43 sec, p = 0.60 V; t = 0.44 sec, p = -0.10 V; t = 0.50 sec, p = -0.10 V). Sucrose, lactose, and Gal-α1,3-Lac concentrations were determined using calibration curves prepared from standards. The mobile phase consisted of degassed 200 mM sodium hydroxide (A) and 18 MΩ-cm water (B), pressurized with inert gas (He) at a flow rate of 0.5 mL/min. The following linear gradient was used for sucrose and lactose detection: t = 0 min, 5:95 (A:B); t = 5 min, 5:95; t = 10 min, 20:80; t = 25 min, 20:80; t = 25 min, 100:0; t = 40 min, 100:0; t = 40 min, 5:95; t = 55 min, 5:95. The linear gradient for Gal-α1,3-Lac detection entails the following steps: t = 0 min, 30:70 (A:B); t = 35 min, 30:70; t = 35 min, 100:0; t = 50 min, 100:0; t = 50 min, 30:70; t = 60 min, 30:70.

#### Intracellular lactose measurement

ATCC 31749/pBQET and ATCC 31749/pBQETY were prepared as described above (α-Gal epitope synthesis). Samples (1 mL) were taken from both reaction vessels at 34 and 45 hours after the start of the synthesis reaction and centrifuged for 5 min at 5,000 × g and 4°C. The supernatant was removed and used for analysis of extracellular lactose and Gal-α1,3-Lac. The cell pellet was washed with 50 mM of Tris-HCl buffer (pH 7.5) and centrifuged a total of three times to remove any residual extracellular lactose. After washing, the cell pellet was resuspended in 600 μL of 50 mM Tris-HCl buffer (pH 7.5). The cells were disrupted using sonication (6 × 10 sec, 1 min rest on ice). Cell debris was removed by centrifugation, and the supernatant containing the intracellular lactose was heated for 10 min in boiling water to denature any proteins. After centrifugation, the sample was diluted 50× and analyzed as described in Carbohydrate analysis to determine the amount of lactose in mmol. The intracellular volume was determined by taking another 1 mL sample and collecting the cell pellet by centrifugation. The cell pellet was washed with DI water and centrifuged three times to remove any residual media. The wet weight of the cell pellet was measured, and then, the cell pellet was dried in an oven at 80°C until a constant dry weight was obtained. The difference in weight between the wet and dry cell pellets was used to calculate the intracellular volume. The intracellular lactose concentration was then calculated using the amount of lactose determined through carbohydrate analysis and the intracellular volume.

#### Curdlan detection

The elimination of curdlan production in ATCC 31749Δ*crdS *was detected using an aniline blue staining method [22]. Candidate colonies were streaked on LB/agar plates containing 0.005% aniline blue. The plates were incubated for 5 days at 30°C, and the stained curdlan (blue) was detected by visual inspection.

## Abbreviations

α1,3-GalT: α1,3-galactosyltransferase; ADP: adenosine diphosphate; ATP: adenosine triphosphate; Crd: curdlan; F6P: fructose-6-phosphate; Fru: fructose; G1P: glucose-1- phosphate; G6P: glucose-6-phosphate; Gal: galactose; GalE: UDP-galactose 4'-epimerase; GalK: galactokinase; GalT: galactose-1-phosphate uridylyltransferase; GalU: glucose-1-phosphate uridylyltransferase; Glc: glucose; IPTG: isopropyl β-D-1-thiogalactopyranoside; Lac: lactose; PEP: phosphoenolpyruvate; UDP: uridine diphosphate; UDPG: uridine diphosphoglucose; UTP: uridine triphosphate

## Competing interests

The authors declare that they have no competing interests.

## Authors' contributions

RRC conceived of the study and participated in the analysis of data and writing of the manuscript. AMR carried out the study and data analysis and participated in the writing of the manuscript. All authors read and approved the final manuscript.
